# Life expectancy and cancer survival in Oncosalud: outcomes over a 15-year period in a Peruvian private institution

**DOI:** 10.3332/ecancer.2021.1336

**Published:** 2021-12-16

**Authors:** Christian Colonio, Luciana Lecman, Joseph A Pinto, Carlos Vallejos, Luis Pinillos

**Affiliations:** 1 División de Riesgos, Oncosalud, AUNA, Lima 15036, Peru; 2 Centro de Investigación Básica y Traslacional, AUNA Ideas, Lima 15036, Peru; 3 Departamento de Radioterapia, Oncosalud-AUNA, Lima 15036, Peru

**Keywords:** cancer, life expectancy, years of life lost, relative survival

## Abstract

**Background:**

There is a large gap in the data on cancer outcomes in Latin America, making it difficult to establish adequate cancer control policies in the region. The aim of our study was to describe the survival, life expectancy estimates and life expectancy changes over time for a large cohort of Peruvian patients insured with Oncosalud, a private healthcare system.

**Patients and methods:**

We evaluated a retrospective cohort of patients diagnosed between 2000 and 2015 in Oncosalud (Lima-Peru). Cases included colon, rectum, stomach, bladder, breast, prostate and non-melanoma skin cancers. Survival was evaluated with the Kaplan–Meier methodology. The standard period life table was used to estimate the excess mortality risks of patients in our cohort compared to the population covered by the Peruvian Superintendence of Banks, Insurance Companies and Pension Funds (SBS). The years of life lost was estimated based on SBS population, matching patients by age and sex.

**Results:**

A large cohort of 7,687 Peruvian cancer patients managed in a 15-year period was eligible. If patients survive 5 years after a cancer diagnosis, life expectancy tends to be close to that of a population without cancer. The number of years of life lost at diagnosis was higher at the youngest ages, steadily decreasing thereafter. During the first years after cancer diagnosis, young patients face a much higher loss in life expectancy than older ones. Patients suffering from colon, rectum, stomach and bladder cancer are the most affected by the years of life lost.

**Conclusion:**

In cancer patients surviving ≥ 5 years, life expectancy becomes similar to that observed in a population with similar socioeconomic characteristics. The estimated survival rate in our cohort is higher than that reported by public cancer registries in Peru. This could be explained by the different socio-economic background and access to specialised cancer care.

## Introduction

The number of cases of cancer is growing around the world as well as the number of deaths [1]. The increasing cost of cancer treatment is a menace for public health, mainly in developing countries. On the other hand, the outcomes and life expectancy of patients with cancer have been improving during the last decades due to early diagnosis, improvement of surgical techniques in radiotherapy, chemotherapy and the development of better targeted therapy and immunotherapy, increasing also the cost of cancer treatment [2].

Life expectancy is an important measure in survival analysis. It is a metric that estimates the average number of years a group of individuals is expected to live at a given age [3]. Life expectancy or gain in life expectancy can provide valuable information to assess the real situation of cancer management and help to establish public policies [3]. Several works have evaluated the changes in survival and life expectancy of cancer patients throughout time [4–6]. Unfortunately, in Peru and in Latin America, there is scarce published data in respect of life expectancy of cancer patients [6–8].

Other measures include relative survival (RS). RS is defined as the ratio of the proportion of observed survivors in a group of cancer patients to the proportion of expected survivors in a comparable group of cancer-free individuals. The RS approach has an advantage over conventional methods in the sense that the cause of death is not required for the estimation [7]. Another useful indicator is the conditional RS, which is calculated as the probability of surviving for an additional period for a patient who has already survived a specified period of time since cancer diagnosis.

In Peru, the Registry of Cancer of Metropolitan Lima and the Ministry of Health (MINSA) collect, analyse and publish data from Lima and Peru as a whole, respectively. Nevertheless, the available data is limited and does not allow to develop a comprehensive survival analysis [8, 9]. In that sense, information from private healthcare institutions is important to measure the impact of cancer in certain subgroups of patients, since this information is not represented in national cancer statistics.

This study aimed to estimate, for the first time in Peru, survival rates, life expectancy and years of life lost estimates of cancer patients since the time of diagnosis for a group of Peruvian people insured with a large private healthcare provider. Both metrics have been analysed by sex and type of diagnosis and compared to those expected from the general Peruvian population.

## Patients and methods

We analysed data from 7,687 patients with histological diagnosis of cancer during the period 2000–2015 (Appendix A, Supplementary Table 1). Patients were affiliated to the prepayment programme of Oncosalud, a Peruvian private healthcare institution specialised in cancer care (with ≈1 million affiliates distributed in several regions of Peru). Patients developing a second primary tumour or patients with *in situ *tumours were excluded. In addition, according to a previously described methodology [4], all cancer-age-sex combinations that had no RS estimates up to 13 years of follow-up and based on less than five cases were excluded from the analysis to obtain consistent estimates. After this selection, the selected cases included colon, rectum, stomach, bladder, breast, non-melanoma skin and prostate cancers.

In Peru, mortality studies are complicated due to the sub register of deaths. It is estimated that by 2016, the coverage for mortality data was <80% [10]. In this study, survival status was obtained from two sources: i) medical and administrative registries of Oncosalud, which involve date of medical attentions, date of renewal of affiliation, etc. Because the coverage of the pre-payment system involves domiciliary palliative care given for free, deaths out of hospitalary facilities are also included in the registers. In addition, patients’ data was cross-checked with ii) database of RENIEC (Peruvian Registry of Identification and Civil Status). The follow-up was conducted until December 31, 2019. Survival probabilities (considering all causes of death) were calculated with the Kaplan–Meier estimator and grouped by sex and cancer type.

Life expectancy is defined as the average number of years a homogeneous group of individuals is expected to live at a certain age [5]. It depends on the complete mortality profile observed in the considered population group, but not on the age structure of the population; it is, therefore, useful as a standardised indicator when comparing overall mortality patterns among different populations [11].

Due to Oncosalud´ affiliates belonging mainly to the A and B socio-economic sectors (related to purchasing power), life tables from the population covered by the Peruvian Superintendence of Banks, Insurance Companies and Pension Funds (SBS) were used as reference in this study to avoid survival bias produced by socio-economic disparities [12]. SBS tables involve data from retired individuals covered by the pension plans (passive members); as well as those that have the right to access a pension benefit if certain events occur (active members). SBS’s mortality tables are a good approximation of Oncosalud affiliates’ profiles because they are based on a population with a similar socioeconomic profile, and it differs from the Peruvian population.

Life expectancy for our cohort of patients was calculated employing the standard period life table method. The period of life table represents mortality rates during a specific period for a certain population and it is used to predict the probability that an individual will die before their next birthday for each age. The older age is set at 110 years according to this table. Life expectancy for our patients was estimated using the proposed methodology by Capocaccia *et al *[5]. First, RS with follow-up up to 13 years was estimated by the period method and the Ederer-2 approach [13].

Estimations considered six-age intervals at time of diagnosis (ages: 0–49, 50–54, 55–59, 60–64, 65–69 and 70–75). The first age interval is wider because of the lower number of cases. The interval-specific RS of cancer patients was then derived from the age at diagnosis and the time since diagnosis. In step two, cancer-specific annual death hazard up to age 110 years, not observable in the current 13-year-long dataset, was estimated for each age interval using the moving average method. Five-year moving average was used to reach age 110 for each cohort of diagnoses. The final step consisted in adding patients’ excess mortality risk to SBS population mortality risk to calculate the life expectancy for cancer patients. For this last calculation, age classes were considered as centred at mid-point (45, 52, 57, 62, 67 and 72 years). Life expectancy for cancer patients was calculated with the same method used for the SBS population. Finally, the years of life lost was estimated as the difference between the life expectancy of the SBS population and life expectancy of cancer patients.

## Results

**Figure 1.**
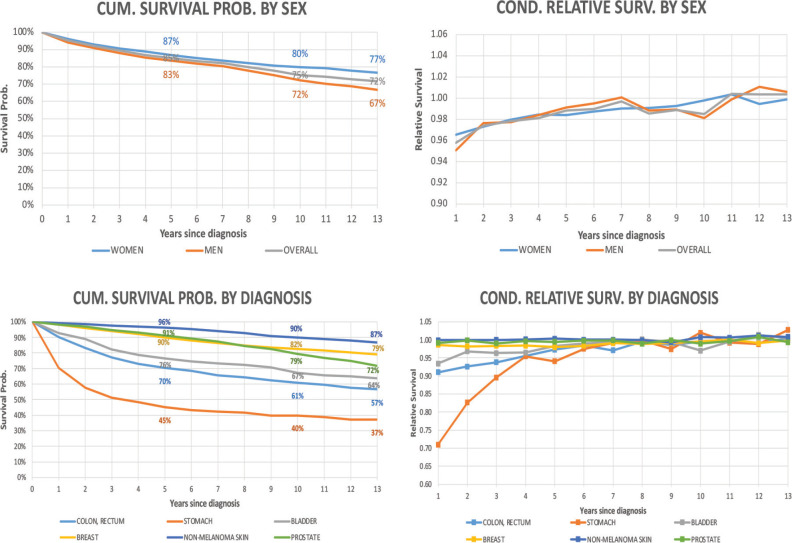
Cumulative survival probability and annual conditional RS according to time since diagnosis by sex and diagnosis. Analysis of Oncosalud’s data, period 2000–2015.

The observed 5-year cumulative survival rate was 85% for the entire cohort, 87% for women and 83% for men, while 10-year survival rate was 75% for all patients, 80% for women and 72% for men (Figure 1). During the first year, the RS probability was 95% and 97% for men and women, respectively. Then it increased steadily along the time, reaching a level close to 100%.

The relative annual survival probability of stomach cancer patients is the lowest among all types of cancer included in the analysis (70% during the first year). For the colon and rectum, the resulting ratio is 90%. Despite this fact, after the seventh year of follow-up, the annual relative rate of patients for all cancer types is close to 100%.

**Figure 2.**
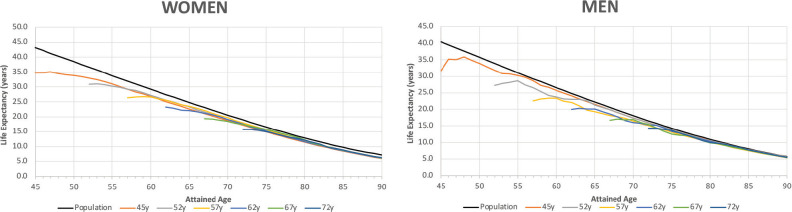
Life expectancy of the general population and cancer patients according to attained age by sex and age at diagnosis. Analysis of Oncosalud’s data, period 2000–2015.

Figure 2 shows the trends in life expectancy of cancer patients by sex according to selected ages at diagnosis, together with the life expectancy of the SBS population. The data points plotted in Figure 2 are displayed in detail in Appendix B, Supplementary Table 2. For both sexes, at the onset of cancer disease, patients showed a dramatic drop in their life expectancy compared to the SBS population. The lost is higher for the youngest age interval and progressively reduces as the age at diagnosis increases. In addition, the number of lost years reduces for both sexes as patients survive the first years of treatment. After 5 years since diagnosis, the difference in life expectancy with respect to SBS individuals reduces to less than 2 years for almost all age intervals.

**Figure 3.**
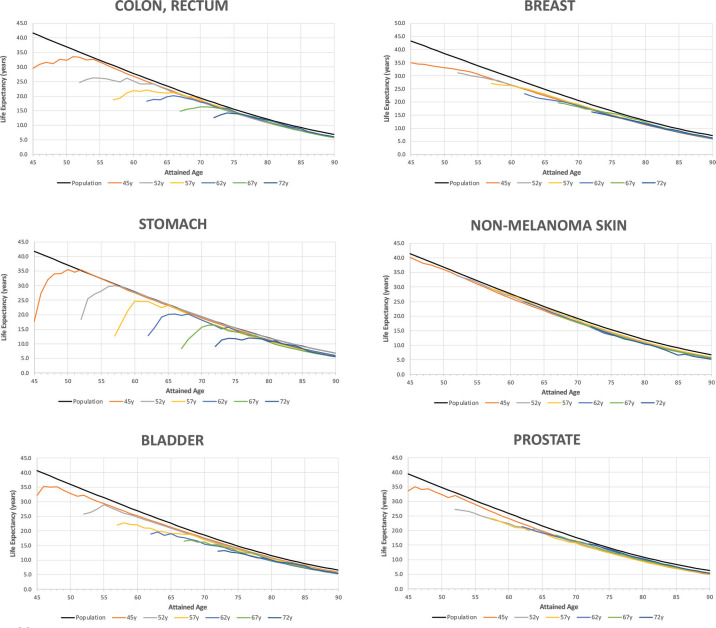
Life expectancy of the general population and of cancer patients according to attained age by cancer type and age at diagnosis. Analysis of Oncosalud’s data, period 2000–2015.

Figure 3 shows the trends in life expectancy of cancer patients according to the selected age at diagnosis compared to the life expectancy of the SBS population by type of diagnosis. Details of Figure 3 for specific data points are reported in Appendix B. According to the life expectancy indicator, two groups of tumours could be identified: The first group was characterised by an initial large drop in patients’ life expectancy compared to the SBS population, followed by a progressive reduction in the gap after the first years of survival (there is a drastic reduction in life expectancy for these patients after the onset of the disease); and the second group showed an initial drop but not as drastic as for the first group. The first group included colon, rectum, stomach and bladder cancers, and the second group included non-melanoma skin, breast and prostate cancer. Non-melanoma skin cancer was the diagnosis with the lowest years of life lost for all ages at diagnosis (less than 2 at any point of attained age). In the first group, patients’ life expectancy ranked from 32.1 (bladder) to 17.6 (stomach) when diagnosed at 45 years old, and patients’ life expectancy ranked from 13.6 (bladder, testis) to 9.2 (stomach) when diagnosed at 72 years old. In the second group, patients’ life expectancy ranked from 40.1 (non-melanoma skin) to 33.7 (prostate) when diagnosed at 45 years old, and patients’ life expectancy ranked from 16.4 (non-melanoma skin) to 15.5 (prostate) when diagnosed at 72 years old.

The years of life lost at age 45 was 23.8 years for stomach cancer (largest), 11.0 years for colon & rectum and 8.6 years for bladder and 7 years for breast cancer. At the age 72, the years of life lost was 6.9 years for stomach, 4.3 years for colon & rectum, 3.5 years for bladder and 2.0 years for breast cancer. The years of life lost varied according to the lethality of the cancer type and age at diagnosis, with more aggressive cancers having a large impact on life expectancy. Over time, all life expectancy curves tend to converge to the SBS population values. In the long term, the patients’ loss of life expectancy with respect to the SBS population depended only on the attained age.

## Discussion

Cancer is one of the main problems of public health in Peru where around 70 thousand of new cases are diagnosed yearly. Prostate, breast and stomach cancers are the most frequent in the Peruvian population. On the other hand, approximately, 35,000 patients with cancer die every year, where stomach and lung cancers have the higher lethality [1].

Life expectancy after cancer diagnosis is increasing as the effectiveness of treatment is improved. This means that several hundred thousand of life years are gained worldwide [11, 14]. Malignancies with the largest proportion of reduction in years of life lost include prostate cancer, non-Hodgkin lymphoma and kidney cancer [11]. Despite the progress achieved, there are still large disparities in results between countries. There is scarce information about long-term outcomes of cancer patients in Latin America [15]. In this study, we provided 15 years data related to a large cohort of patients from Peru.

Our study has some limitations, including the influence of the changing landscape of cancer management during the study period is missing; our patients’ population belongs mainly to the A and B socio-economic sectors and their oucomes are not representative of the general Peruvian population. Socio-economic disparities between socio-economic strata in Peru could lead to bias in life expectancy and RS estimations [15]. On the other hand, our cohort of patients had complete access to state-of-the-art cancer treatments while annual cancer screening is offered for free to healthy affiliates. Unfortunately, barriers to specialised cancer care and delayed time to access treatment in the public system affect negatively the evolution of the disease [16]. Other limitation of our work has been it doesn’t include the scenario of COVID-19 pandemic that increases the disparities in access to healthcare between socio-economic strata and impacts directly in the survival probability of patients in Peru [17].

**Figure 4.**
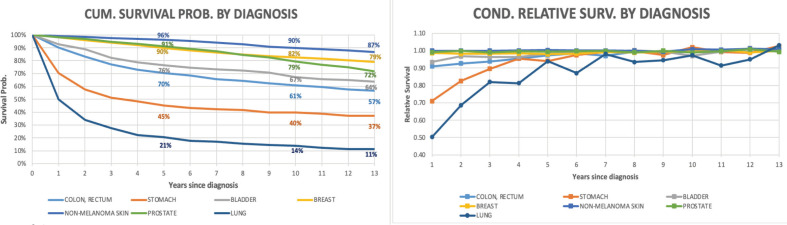
Cumulative survival probability and annual conditional RS by diagnosis including all ages. Analysis of Oncosalud’s data, period 2000–2015.

In addition to the six types of cancers selected for this analysis in this work, we present the results for lung cancer (due to its importance) despite this malignancy did not meet the needed criteria. In Figure 4, lung cancer was the diagnosis which presented the lowest survival rate; nevertheless, after 6 years since diagnosis, the conditional RS rates are over 90%. Life expectancy estimate for this diagnosis was not possible to calculate due to the lack of data for all age intervals.

In our study, we observed a large number of years of life lost after diagnosis, independently of sex or age at diagnosis in comparison to the life expectancy of a similar Peruvian population without cancer. In patients aged 57–62 years old, the life expectancy after 5 years of diagnosis is remarkably similar to the reference population. Life expectancy is similar to the reference population 1 year after diagnosis in patients aged 72 or older. These findings are similar to other studies with similar aims [4]. The life expectancy for our patients was higher than the results described by Botta *et al *[4] for Italian patients and for the study by Capocaccia *et al *[5] conducted in patients with colon, breast and testicular cancers from the Surveillance, Epidemiology, and End Results cohort.

The results of our study should be interpreted carefully because it represents to a special cohort without the barriers of access to drugs that is common in the Peruvian public system [18]. We observed some differences in 5-year survival rate in comparison to data of CONCORD-3 programme evaluating data from the Metropolitan Lima Cancer Registry. Five-year survival rate was 90% in our cohort versus 84% in the CONCORD-3 study; in addition, survival rate for our patients with colon and rectum cancer was 70% versus 59% for colon and 54.8% for rectum patients in the study CONCORD-3. High rates of survival for breast cancer in Peru reported by the CONCORD-3 study (similar to several developed countries in that report) could be attributed to a bias in retrieving data for survival status by the coverage of mortality data as explained in the methods section.

Survivorship care is an important research topic [19]; specific detailed estimates and projections of the number of people surviving after being diagnosed with different cancer types and real-world estimates of the impact of cancer on specific populations are particularly relevant to policy makers. Changes in life expectancy during the disease can provide a different and complementary point of view for cancer cure with respect to the RS-based criteria [20]. On the other hand, with an increasing life expectancy of cancer patients in Peru, it is important to develop more survivorship care plans and policies to protect the reincorporation of cancer survivors to the economic activity [21].

There is an urgent need in Peru for updated statistical data to assess current results of the deployment of activities of our national cancer control plan, and in this way to evaluate if we are achieving the proposed strategic aims. The current technical document of the national comprehensive cancer care plan (2020–2024) uses mortality data of 2016 obtained from the death registry of the Ministry of Health (MINSA) where data from private institutions is invisible [22]. This scenario is difficult to obtain precise estimation of cancer mortality, survival and life expectancy of patients. Plans to share epidemiological data from cancer patients should be a priority to strength the strategies of cancer control. More initiatives in research of cancer epidemiology should improve this landscape.

## Conclusion

In conclusion, we have quantified the impact of cancer in life expectancy for our cohort of patients. Future studies are needed to evaluate the impact of the introduction and massification of new therapies such as tyrosine kinase inhibitors and immunotherapy in the lives of our patients. With a few more years of data, we expect to expand this study to evaluate the progress in other malignancies that did not meet the criteria to be included in the present study.

## Funding

None.

## Conflicts of interest statement

The authors declare they have no potential conflicts of interests with this research.

## Authors’ contributions

Conceptualisation: CV, LP

Data curation: CC, LL

Formal analysis: CC, LL, JAP

Methodology: CC, LL, JAP

Project administration: LL, CV, LP

Validation: JAP

Visualisation: CC

Writing – original draft: All authors

Writing – review & editing: All Authors.

## Figures and Tables

**Table 1.** Life expectancy and years of life lost of all cancer patients with respect to the age-matched cancer-free population at specific time points after diagnosis (0, 1, …, 5, 10 and 13 years) by sex and age at diagnosis.

	Years since Diagnosis	Life expectancy (years of life lost)					
		Age at diagnosis					
Sex		45	52	57	62	67	72
Women	0	34.7 (7.2)	30.9 (4.6)	26.3 (4.6)	23.2 (3.2)	19.4 (2.7)	15.7 (2.3)
	1	34.7 (6.2)	31.0 (3.5)	26.6 (3.4)	22.8 (2.7)	19.2 (2.0)	15.7 (1.5)
	2	34.9 (5.1)	30.7 (2.9)	26.7 (2.4)	22.2 (2.4)	18.8 (1.5)	15.5 (0.9)
	3	34.4 (4.6)	30.2 (2.4)	26.6 (1.6)	22.1 (1.7)	18.4 (1.1)	14.9 (0.7)
	4	34.1 (4.0)	29.9 (1.9)	25.9 (1.4)	21.6 (1.2)	17.8 (1.0)	14.4 (0.5)
	5	33.8 (3.4)	29.1 (1.7)	25.6 (0.9)	21.1 (0.9)	17.3 (0.5)	13.7 (0.3)
	10	31.0 (1.5)	25.5 (0.8)	22.1 (0.0)	17.5 (0.4)	14.4 (−0.3)	10.7 (−0.0)
	13	28.2 (1.5)	23.3 (0.3)	19.7 (−0.1)	15.1 (0.3)	12.2 (−0.3)	8.8 (0.0)
Men	0	31.6 (7.2)	27.1 (5.3)	22.5 (5.0)	20.0 (3.4)	16.7 (2.4)	14.2 (1.2)
	1	35.1 (2.7)	27.8 (3.7)	23.1 (3.5)	20.3 (2.2)	17.1 (1.3)	14.3 (0.4)
	2	35.0 (1.9)	28.1 (2.5)	23.3 (2.5)	20.1 (1.6)	16.8 (0.8)	13.9 (0.0)
	3	35.8 (0.2)	28.5 (1.2)	23.3 (1.6)	20.0 (0.8)	16.9 (−0.1)	13.7 (−0.3)
	4	34.8 (0.3)	27.3 (1.5)	22.4 (1.6)	19.3 (0.7)	16.0 (0.0)	12.8 (−0.2)
	5	33.8 (0.4)	26.5 (1.4)	22.0 (1.2)	18.5 (0.6)	15.4 (−0.0)	12.2 (−0.1)
	10	30.2 (−0.5)	23.0 (0.5)	18.1 (0.9)	15.3 (0.0)	12.0 (−0.0)	9.37 (−0.2)
	13	27.2 (−0.2)	21.2 (−0.1)	16.4 (0.2)	13.3 (−0.0)	9.90 (0.2)	7.7 (−0.1)

## Appendix A

**Supplementary Table 1.** Cases for each type of diagnosis and sex – Oncosalud’s database, period 2000–2015.

Diagnosis	Female	Male	Total
Colon, rectum	487	435	922
Stomach	218	249	467
Bladder	195	360	555
Breast	2,577	10	2,587
Non-melanoma skin	842	678	1,520
Prostate	0	1,636	1,636
**Total**	4,319	3,368	7,687

## Appendix B

**Supplementary Table 2.** Life expectancy and years of life lost of all cancer patients with respect to the diagnosis-matched cancer-free population at specific time points after diagnosis (0, 1, …, 5, 10 and 13 years) by sex and age at diagnosis.

	Years since diagnosis	Life expectancy (years of life lost)					
		Age at diagnosis					
Cancer type		45	52	57	62	67	72
Colon, rectum	0	29.5 (11.1)	24.6 (9.1)	18.8 (11.1)	18.3 (6.9)	14.8 (5.8)	12.7 (4.3)
	1	30.9 (8.8)	25.7 (7)	19.4 (9.5)	18.8 (5.5)	15.5 (4.2)	13.5 (2.7)
	2	31.6 (7.1)	26.3 (5.5)	21.1 (6.9)	18.7 (4.7)	15.8 (3.1)	14.2 (1.3)
	3	31.1 (6.7)	26.1 (4.8)	21.9 (5.3)	19.8 (2.8)	16.3 (1.8)	14 (0.6)
	4	32.6 (4.2)	25.9 (4.1)	21.7 (4.6)	20.1 (1.6)	16.2 (1.1)	13.9 (0)
	5	32.2 (3.7)	25.4 (3.7)	22.1 (3.3)	19.8 (1.1)	16.1 (0.5)	13.2 (0.1)
	10	31.7 (-0.3)	24.1 (0.5)	20.8 (0.3)	16.5 (0.3)	13.3 (-0.4)	10.5 (-0.5)
	13	28.7 (0)	22.2 (0)	18.7 (0)	14.8 (-0.2)	11.3 (-0.4)	8.8 (-0.5)
							
Stomach	0	17.6 (23.8)	18.3 (17.1)	12.7 (16.4)	12.9 (12.6)	8.5 (11.2)	9.2 (6.9)
	1	27.3 (13.1)	25.4 (9)	17.2 (11)	15.8 (8.8)	11.5 (7.4)	11.3 (4.1)
	2	31.9 (7.6)	27 (6.5)	21.4 (5.9)	19.2 (4.6)	13.6 (4.5)	11.9 (2.8)
	3	34 (4.6)	28.1 (4.5)	24.6 (1.8)	20.1 (2.8)	15.7 (1.6)	11.8 (2.1)
	4	34.1 (3.5)	29.6 (2.1)	24.5 (1)	20.2 (1.8)	16.4 (0.1)	11.3 (1.9)
	5	35.6 (1.1)	29.9 (0.9)	24.5 (0.1)	19.8 (1.4)	16.4 (-0.5)	12 (0.6)
	10	32.4 (-0.3)	26 (0.3)	21.2 (-0.8)	16.2 (1)	12.9 (-0.7)	10.1 (-0.6)
	13	29.4 (0)	23.4 (0.2)	18.2 (-0.1)	14.7 (0.2)	10.8 (-0.4)	8.4 (-0.5)
Bladder	0	32.1 (8.6)	25.7 (8.4)	22.1 (7.1)	18.9 (4.9)	16.5 (3.7)	13 (3.5)
	1	35.2 (4.5)	26.3 (6.9)	22.7 (5.6)	19.6 (3.4)	16.8 (2.6)	13.2 (2.6)
	2	35 (3.8)	27.5 (4.7)	22.2 (5.2)	18.5 (3.6)	16.1 (2.5)	12.7 (2.3)
	3	35.2 (2.5)	29 (2.3)	22.1 (4.4)	19 (2.3)	16.2 (1.6)	12.5 (1.8)
	4	33.9 (3)	28 (2.4)	21.1 (4.5)	18 (2.5)	15.3 (1.7)	12 (1.6)
	5	32.9 (3.1)	27 (2.5)	20.9 (3.9)	17.6 (2)	15.2 (1.1)	11.3 (1.6)
	10	29.3 (2.1)	23.2 (1.9)	18.8 (1.7)	14.8 (0.8)	12.5 (0.1)	9.1 (0.6)
	13	26.8 (1.9)	20.9 (1.6)	17 (1.1)	12.9 (0.6)	9.8 (0.8)	8 (0)
Breast	0	35 (7)	31.1 (4.5)	27 (3.9)	23.1 (3.4)	19.7 (2.6)	16.2 (2)
	1	34.4 (6.6)	30.6 (4)	26.6 (3.4)	22.2 (3.5)	19.4 (2)	15.7 (1.7)
	2	34.4 (5.7)	30 (3.7)	26.4 (2.7)	21.5 (3.3)	18.7 (1.9)	15.2 (1.4)
	3	33.8 (5.3)	29.6 (3.2)	26.2 (2)	21.1 (2.8)	18.2 (1.6)	14.6 (1.2)
	4	33.4 (4.8)	29.2 (2.6)	25.6 (1.7)	20.7 (2.3)	17.4 (1.5)	14 (1.1)
	5	33.1 (4.1)	28.6 (2.3)	25.3 (1.1)	20.3 (1.9)	17.2 (0.9)	13.6 (0.7)
	10	30.6 (2)	24.9 (1.5)	21.4 (0.7)	17.2 (0.8)	14.5 (-0.3)	10.6 (0.2)
	13	27.9 (1.9)	23 (0.8)	19.2 (0.3)	14.8 (0.7)	12.2 (-0.1)	9 (0)
Non-melanoma skin	0	40.1 (-0.2)	33.9 (0)	29.5 (0.1)	25 (0.1)	20.2 (0)	16.4 (0.7)
	1	39.1 (-0.1)	33.1 (-0.1)	28.8 (-0.1)	24.1 (0)	19.5 (-0.1)	15.5 (0.8)
	2	38.1 (0)	32.6 (-0.5)	28.1 (-0.3)	23.3 (0)	18.7 (0)	14.6 (1)
	3	37.6 (-0.5)	31.6 (-0.4)	27.2 (-0.3)	22.4 (0.1)	17.9 (0)	13.8 (1)
	4	36.8 (-0.5)	30.6 (-0.4)	26.5 (-0.5)	21.5 (0.1)	17 (0)	13 (1.1)
	5	36 (-0.7)	29.6 (-0.3)	25.5 (-0.3)	20.6 (0.1)	16.2 (0)	12.2 (1.2)
	10	31 (-0.3)	24.9 (0)	21.2 (-0.3)	16.1 (0.6)	12.5 (0)	9.2 (0.9)
	13	28 (-0.1)	22.5 (-0.1)	18.6 (-0.2)	13.5 (1)	10.5 (0.1)	6.7 (1.7)
Prostate	0	33.7 (4)	27.3 (5)	24.4 (3.1)	21.3 (2.1)	18.5 (0.6)	15.5 (0)
	1	35.1 (1.6)	26.9 (4.5)	23.8 (2.8)	20.7 (1.8)	17.8 (0.5)	14.8 (-0.1)
	2	34.1 (1.7)	26.6 (3.9)	22.9 (2.8)	19.9 (1.8)	16.9 (0.7)	14.2 (-0.1)
	3	34.3 (0.6)	25.9 (3.7)	21.9 (3)	19.2 (1.6)	16.5 (0.3)	13.6 (-0.2)
	4	33.3 (0.6)	24.9 (3.8)	21 (3)	18.7 (1.3)	15.7 (0.4)	12.9 (-0.3)
	5	32.3 (0.7)	24.2 (3.6)	20.9 (2.2)	18 (1.2)	15.1 (0.3)	12.2 (-0.1)
	10	29 (-0.5)	21.1 (2.3)	17.3 (1.7)	15.2 (0.1)	11.9 (0)	9.2 (-0.1)
	13	26 (-0.1)	19.5 (1.4)	15.8 (0.8)	13.3 (-0.1)	9.8 (0.2)	7.6 (0)
